# Inflammatory Indices as Markers of Vascular and Organ Involvement in Behçet’s Disease

**DOI:** 10.3390/jcm15041535

**Published:** 2026-02-15

**Authors:** Jehat Kılıç, Gülşah Yamancan, Yusuf Doğan, Sümeyye Şahin, İbrahim Gündüz, Ömer Faruk Alakuş, Burak Öz, Ahmet Karataş, Süleyman Serdar Koca

**Affiliations:** 1 Department of Rheumatology, Fırat University Elazığ, Elazığ 23119, Turkey; gulsahaydn@windowslive.com (G.Y.); yusuf.230@hotmail.com (Y.D.); sumeyye_balta@hotmail.com (S.Ş.); boz@firat.edu.tr (B.Ö.); a.karatas@firat.edu.tr (A.K.); sskoca@firat.edu.tr (S.S.K.); 2 Department of Rheumatology, Gazi Yaşargil Training and Research Hospital, Diyarbakır 21090, Turkey; abrahim724gunduz@hotmail.com (İ.G.); omerfaruk01@gmail.com (Ö.F.A.)

**Keywords:** behçet’s disease, ınflammatory burden ındex, erythrocyte sedimentation rate, vascular involvement, mortality

## Abstract

**Background:** Behçet’s disease is a multisystem inflammatory disorder with a variable clinical course. This study evaluated the association between inflammatory indices, clinical involvement, and mortality. **Methods:** This retrospective study included 444 patients with BD. Clinical characteristics and laboratory data were systematically retrieved from electronic medical record system. Inflammatory indices (NLR, PLR, SII) were calculated to reflect systemic inflammation. In addition, CRP-based composite indices (IBI-NLR and IBI-SII) were derived to integrate cellular and acute-phase inflammatory responses. Disease manifestations, major organ involvement, comorbidities, and mortality were recorded to comprehensively assess disease burden and clinical outcomes. **Results:** In multivariable analysis, vascular involvement was associated with increased ESR level (OR = 1.013, 95% CI: 1.002–1.024, *p* = 0.018), and male sex (OR = 3.22, 95% CI: 1.83–5.67, *p* < 0.001;). ROC analysis showed the highest discriminatory performance for vascular involvement, with IBI-NLR (AUC = 0.624, *p* < 0.001), IBI-SII (AUC = 0.609, *p* = 0.001) and NLR (AUC = 0.597, *p* = 0.004). Moreover, NLR (AUC = 0.571, *p* = 0.017), IBI-NLR (AUC = 0.576, *p* = 0.010), and IBI-SII (AUC = 0.562, *p* = 0.036) had modest discrimination for major organ involvement. In contrast, inflammatory indexes were not predictive for mortality (*p* > 0.05 for all). Mortality was independently associated with higher creatinine (OR = 1.086, *p* = 0.048), higher ESR (OR = 1.023, *p* = 0.046), and lower uric acid levels (OR = 0.454, *p* = 0.002). **Conclusions:** Inflammatory indices may not predict mortality in BD but can help identify vascular and major organ involvement. Male sex and ESR level are associated more severe disease, while mortality is associated with renal dysfunction and systemic inflammation in BD.

## 1. Introduction

Behçet’s disease (BD) is a systemic autoimmune disorder characterized by recurrent oral aphthous ulcers, genital ulcers, uveitis, and a marked tendency toward vascular thrombosis. It can involve multiple organ systems, leading to a wide spectrum of clinical manifestations. The disease is most prevalent among populations living along the historical Silk Road, extending from the Mediterranean basin to East Asia, suggesting a strong interaction between genetic susceptibility and environmental factors [1,2,3]. Diagnosis is primarily clinical and is based on established classification criteria, most commonly the International Criteria for Behçet’s Disease (ICBD), which incorporate recurrent oral ulcers together with genital ulcers, ocular lesions, skin manifestations, vascular involvement, neurological findings, and a positive pathergy test [4].

A wide range of laboratory-based parameters have been used to assess inflammatory activity in systemic and inflammatory diseases. Among these, composite hematologic indices such as the neutrophil-to-lymphocyte ratio (NLR), platelet-to-lymphocyte ratio (PLR), systemic immune-inflammation index (SII), and C-reactive protein-based inflammatory burden indices, including IBI-NLR and IBI-SII, have gained increasing attention [5,6,7]. These markers integrate information from different components of the inflammatory response and have been shown to reflect disease activity, organ involvement, and prognosis across various immune-mediated and inflammatory conditions [7].

In this study, we aimed to comprehensively evaluate the associations between these inflammatory indices and clinically relevant outcomes in BD, specifically vascular involvement, major organ involvement, and mortality. By examining both conventional hematologic ratios and CRP-based composite inflammatory burden indices, we sought to determine their potential value in reflecting disease severity, organ involvement, and prognostic risk.

## 2. Materials and Methods

### 2.1. Study Overview

This retrospective study included 444 patients diagnosed with Behçet’s disease who were followed as inpatients and outpatients at the Department of Rheumatology, Fırat University Hospital (Elazığ/Turkey). Clinical and laboratory data were retrieved from the hospital electronic medical record system using a standardized data extraction process (ENLIL, Version 3.2; Enlil Software Inc., Ankara, Türkiye). The study was conducted in accordance with the principles of the Declaration of Helsinki. Ethical approval was obtained from the local ethics committee on 27 November 2025 (approval number: 17–34). Due to the retrospective nature of the study, the requirement for written informed consent was waived by the ethics committee.

### 2.2. Data Collection

Laboratory parameters retrieved for analysis included white blood cell count, neutrophil count, lymphocyte count, platelet count, hemoglobin level, serum creatinine, urea, alanine aminotransferase, gamma-glutamyl transferase (GGT), uric acid, CRP, and erythrocyte sedimentation rate (ESR). Because reference ranges may vary between laboratories, the normal reference values used at our institution were as follows: C-reactive protein (CRP) <5 mg/L and erythrocyte sedimentation rate (ESR) <20 mm/h. Clinical and laboratory data were collected during the disease course at routine follow-up visits and/or hospital admissions. In addition to individual laboratory parameters, several inflammatory indices were calculated to better reflect systemic inflammatory burden. The NLR was calculated as the ratio of absolute neutrophil count to absolute lymphocyte count, while the PLR was calculated by dividing the platelet count by the lymphocyte count. The systemic immune-inflammation index was calculated using the formula neutrophil count multiplied by platelet count divided by lymphocyte count [8,9,10]. There are no universally accepted reference ranges or cutoff values for inflammatory indices such as NLR, PLR, IBI-NLR, and IBI-SII. Therefore, these indices were analyzed as continuous variables, and optimal cutoff values were determined using receiver operating characteristic (ROC) curve analysis.

To further integrate acute-phase response into composite inflammatory measures, CRP-based inflammatory burden indices were derived. The IBI-NLR was calculated by multiplying CRP by the neutrophil-to-lymphocyte ratio, and the IBI-SII was calculated by multiplying C-reactive protein by the systemic immune-inflammation index. These indices were used to capture both cellular inflammatory activity and acute-phase reactant levels in a single composite measure [11].

### 2.3. Inclusion Criteria

Patients were included if they were ≥18 years of age, had a confirmed diagnosis of BD according to established classification criteria (ICBD or ISG), were followed at our institution, and had available laboratory data (complete blood count, CRP and/or ESR) allowing calculation of inflammatory indices (NLR, PLR, SII, IBI-NLR, and IBI-SII).

### 2.4. Exclusion Criteria

Patients were excluded if they were <18 years old, had an unconfirmed BD diagnosis, missing key laboratory data, or had conditions likely to markedly influence inflammatory markers independent of BD, including active infection, malignancy, hematologic disorders, or pregnancy. Duplicate records were also excluded.

Variables related to the diagnosis of BD included mucocutaneous findings (oral aphthous ulcers, genital ulcers, erythema nodosum-like lesions (ELL), and acneiform lesions, and pathergy test results when available. Organ involvement and disease-related complications were assessed by recording vascular involvement, uveitis, articular involvement, gastrointestinal involvement, and neurological manifestations. Neurological involvement referred to clinically and/or radiologically confirmed neuro-Behçet’s disease. Vascular involvement included venous thrombosis and arterial involvement attributable to Behçet’s disease. MACE comprised myocardial infarction, stroke, or cardiovascular death.

*Statistical Analysis:* The distribution of continuous variables was assessed using the Kolmogorov–Smirnov test. Data with a normal distribution are presented as mean ± standard deviation, whereas non-normally distributed variables are reported as median (minimum–maximum). Comparisons between groups were performed using Student’s t-test for normally distributed continuous variables and the Mann–Whitney U test for non-normally distributed variables. ROC curve analyses were conducted to evaluate the discriminatory performance of the studied parameters. Statistical analyses were performed using SPSS version 26.0 (IBM Corp., Armonk, NY, USA). Comparisons of ROC curves were carried out using the DeLong test with the Jamovi statistical software (Version 2.7.16; The Jamovi Project, Sydney, Australia). A two-sided *p* value < 0.05 was considered statistically significant.

## 3. Results

The study population consisted of 178 women (40.1%) and 266 men (59.9%). Mucocutaneous manifestations were common in both sexes, with no significant differences in oral aphthae, genital ulcers, uveitis, ELL acneiform lesions, or neurologic involvement. In contrast, vascular involvement and major organ involvement were significantly more frequent in male patients (both *p* = 0.001), while mortality was low and comparable between sexes (*p* = 0.776) (Table 1).

Patients with vascular involvement exhibited a significantly higher inflammatory burden compared with those without vascular involvement (Table 2). White blood cell and neutrophil counts were significantly increased in the vascular involvement group (*p* = 0.006 and *p* = 0.004, respectively), whereas lymphocyte, platelet, and hemoglobin levels were comparable between groups (*p* > 0.05 for all). Serum creatinine was modestly but significantly higher in patients with vascular involvement (*p* = 0.018), while urea levels did not differ (*p* = 0.173). Markers of systemic inflammation, including CRP (*p* = 0.001), ESR (*p* = 0.009), GGT (*p* = 0.010), and uric acid (*p* = 0.003), were consistently elevated in patients with vascular involvement. Among inflammatory indices, both NLR (*p* = 0.004) and SII (*p* = 0.044) were significantly higher, whereas PLR showed no significant difference (*p* = 0.319). Notably, CRP-based composite indices demonstrated the strongest associations, with markedly higher IBI-NLR (*p* = 0.0001) and IBI-SII (*p* = 0.001) values in the vascular involvement group.

Patients with major organ involvement demonstrated a higher inflammatory and renal burden compared with those without major organ involvement (Table 3). White blood cell and neutrophil counts were significantly higher in patients with major organ involvement (*p* = 0.003 and *p* = 0.010, respectively), whereas lymphocyte, platelet, and hemoglobin levels were comparable between groups (*p* > 0.05 for all). Renal parameters differed significantly, with higher serum creatinine (*p* = 0.0001) and urea levels (*p* = 0.004) in patients with major organ involvement. Markers of systemic inflammation were partially elevated, including GGT (*p* = 0.005), uric acid (*p* = 0.010), and ESR (*p* = 0.030), while CRP levels did not differ significantly (*p* = 0.136). Among inflammatory indices, NLR was significantly higher (*p* = 0.017), whereas PLR and SII showed no significant differences (*p* = 0.911 and *p* = 0.234, respectively). Notably, CRP-based composite indices were significantly elevated, with higher IBI-NLR (*p* = 0.010) and IBI-SII (*p* = 0.036), indicating an increased composite inflammatory burden in patients with major organ involvement.

Comparisons between survivors and non-survivors are summarized in Table 4. Overall, most hematologic, biochemical, and inflammatory parameters—including white blood cell, neutrophil and lymphocyte counts, platelet count, hemoglobin, urea, liver enzymes, CRP, and composite inflammatory indices (NLR, PLR, SII, IBI-NLR, and IBI-SII)—did not differ significantly between groups (all *p* > 0.05). In contrast, non-survivors had higher serum creatinine levels and elevated ESR compared with survivors (*p* = 0.007 and *p* = 0.003, respectively), while uric acid levels were significantly lower in the non-survivor group (*p* = 0.001).

**Figure 1:**
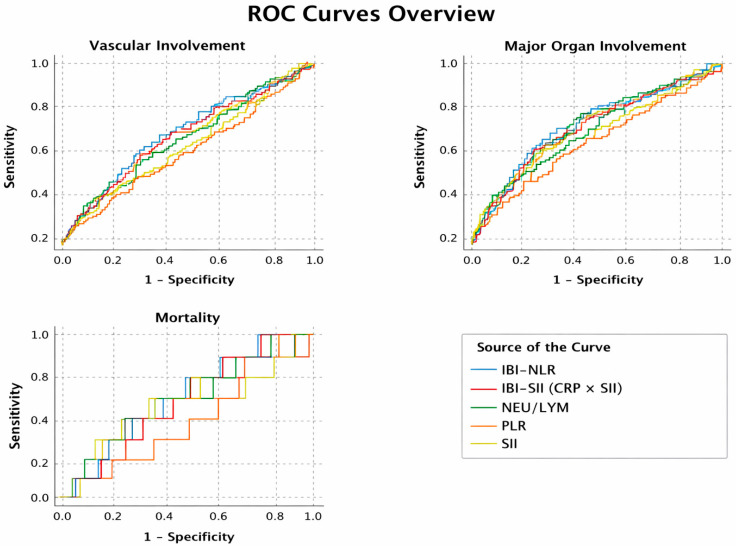
Receiver Operating Characteristic Curves of Inflammatory Indices for Vascular Involvement, Major Organ Involvement, and Mortality in Behçet Disease.

The discriminatory performance of inflammatory indices for mortality was poor, with no marker showing significant predictive ability (AUC range: 0.462–0.533; all *p* > 0.05). For major organ involvement, NLR demonstrated modest but significant discrimination (AUC = 0.571; *p* = 0.017), while CRP-based composite indices showed slightly improved performance (IBI-NLR AUC = 0.576, *p* = 0.010; IBI-SII AUC = 0.562, *p* = 0.036). In contrast, PLR and SII alone were not significant. The strongest associations were observed for vascular involvement, where NLR, SII, and composite indices demonstrated significant discrimination. Among all markers, IBI-NLR achieved the highest AUC (0.624; *p* < 0.001), followed by IBI-SII (AUC = 0.609; *p* = 0.001), whereas PLR was not predictive (Figure 1, Table 5).

For mortality, NLR, PLR, SII, and IBI-SII demonstrated similar sensitivities (all 84.6%), while IBI-NLR showed the highest sensitivity (92.3%). Specificity was low across all markers, and the Youden indices were uniformly modest (~0.12–0.13), indicating limited and comparable discriminatory performance. In predicting major organ involvement, NLR achieved the highest sensitivity (92.0%), whereas IBI-SII showed the greatest specificity (29.4%) and the highest Youden index (0.055). For vascular involvement, NLR and IBI-NLR exhibited high sensitivities (93.3% and 92.2%, respectively); however, IBI-SII provided the best overall discrimination, with the highest Youden index (0.124) owing to a more balanced sensitivity (84.6%) and specificity (27.8%) (Table 6).

In univariate logistic regression analysis, gender, vascular involvement, major organ involvement, hemoglobin level, platelet count, and inflammatory indices (NLR, PLR, SII, IBI-NLR, and IBI-SII) were not significantly associated with the outcome (all *p* > 0.05). In contrast, uric acid, creatinine, and erythrocyte sedimentation rate (ESR) showed significant associations. In multivariable analysis, uric acid remained independently associated with a lower odds of the outcome (OR = 0.454, 95% CI: 0.278–0.741; *p* = 0.002), while creatinine was associated with an increased risk (OR = 1.086, 95% CI: 1.001–1.179; *p* = 0.048). ESR also emerged as an independent predictor, with higher values modestly increasing the odds of the outcome (OR = 1.023, 95% CI: 1.000–1.046; *p* = 0.046) (Table 7).

In univariate logistic regression analysis, male gender, ESR, and several inflammatory indices—including NLR, SII, IBI-NLR, and IBI-SII—were significantly associated with the outcome. Male sex was associated with a more than twofold increase in risk (OR = 2.45, 95% CI: 1.58–3.82; *p* = 0.0001). Higher ESR and inflammatory index values were also linked to increased odds, whereas uric acid, creatinine, platelet count, hemoglobin, and PLR were not significantly associated. In the multivariable model, male gender remained a strong independent predictor, with a more than threefold higher odds of the outcome (OR = 3.22, 95% CI: 1.83–5.67; *p* = 0.0001). ESR also remained independently associated, with higher values modestly increasing risk (OR = 1.013, 95% CI: 1.002–1.024; *p* = 0.018). In contrast, IBI-SII lost its significance after adjustment, suggesting that its effect may be mediated by other covariates in the model (Table 8).

In univariate logistic regression analysis, male gender was significantly associated with the outcome, with males having more than a twofold higher odds compared with females (OR = 2.45, 95% CI: 1.58–3.82; *p* = 0.001). ESR was also significantly associated, indicating increased odds with rising inflammatory activity (OR = 1.013, 95% CI: 1.004–1.023; *p* = 0.007). Among inflammatory indices, NLR and IBI-SII showed significant associations, while SII demonstrated a borderline association. Uric acid, creatinine, platelet count, hemoglobin, PLR, and IBI-NLR were not significantly associated with the outcome. In the multivariable model, male gender remained an independent predictor, with a more than twofold increase in odds (OR = 2.43, 95% CI: 1.55–3.80; *p* = 0.0001). ESR also remained independently associated, albeit with a modest effect size (OR = 1.011, 95% CI: 1.001–1.023; *p* = 0.039). In contrast, the association observed for IBI-SII in univariate analysis did not persist after adjustment (Table 9).

## 4. Discussion

In the present study, inflammatory markers were not independently associated with mortality, suggesting that these indices may have limited value in predicting survival in BD. Instead, they appeared to be more informative for vascular and major organ involvement, supporting the concept that inflammatory indices primarily reflect disease severity rather than fatal outcome. This is consistent with previous observations that vascular involvement represents one of the most severe manifestations of BD, particularly in male patients [11]. Notably, CRP-based composite inflammatory indices outperformed conventional hematologic ratios, likely because they integrate both cellular inflammation and acute-phase response, providing a more comprehensive measure of systemic inflammatory burden. Furthermore, the consistent association of male sex and elevated ESR with more severe disease aligns with the established role of sustained systemic inflammation in the pathogenesis of vascular and organ involvement in BD [12].

Vascular involvement has a major impact on the clinical course of BD, affecting disease severity, therapeutic decisions, and long-term prognosis, and is closely associated with increased mortality [13]. In our study, the superior performance of IBI-NLR and IBI-SII compared with other inflammatory markers may be explained by their ability to better reflect the overall systemic inflammatory burden. These composite indices integrate C-reactive protein, a key acute-phase reactant, with cellular inflammatory markers such as NLR and SII, thereby capturing both acute-phase and cellular components of inflammation. This integrated approach may provide a more comprehensive representation of the inflammatory processes underlying vascular involvement in BD [2].

Major organ involvement was defined as the presence of neurological involvement (neuro-Behçet’s disease), vascular involvement, or MACE. Neurological involvement referred to clinically and/or radiologically confirmed neuro-Behçet’s disease. Vascular involvement included venous thrombosis and arterial involvement attributable to Behçet’s disease. MACE comprised myocardial infarction, stroke, or cardiovascular death. Although the AUC values of NLR, IBI-NLR, and IBI-SII were moderate, these indices demonstrated statistically significant discriminatory ability for major organ involvement. While these markers do not appear to be suitable as stand-alone diagnostic tools, they may serve as supportive inflammatory parameters in clinical assessment. Moreover, rather than being used for population screening, these indices may have greater utility in risk stratification, helping to identify patients at higher risk for severe organ involvement.

The low mortality rate observed in our cohort may have limited the ability to demonstrate the prognostic value of the studied inflammatory parameters for mortality. In BD, mortality is generally driven by renal dysfunction, cumulative organ damage, and associated comorbidities, rather than by markers reflecting acute inflammatory activity. Therefore, the inflammatory indices evaluated in this study may be more informative for assessing current disease activity and inflammatory burden than for predicting long-term mortality outcomes [14]. In our study, male sex and ESR emerged as strong parameters associated with mortality. BD is known to show a male predominance, and male sex has consistently been linked to a more severe disease course and higher mortality risk. The stronger association of ESR with mortality—compared with CRP-based indices—may reflect the role of persistent, underlying chronic inflammation rather than acute inflammatory activity in driving long-term adverse outcomes in BD [3,14].

The inflammatory indices evaluated in this study are inexpensive, widely available, and easily derived from routine laboratory parameters, making them suitable for everyday clinical practice. Although these markers lack sufficient accuracy to be used as standalone diagnostic or prognostic tools, they can provide supportive and complementary information when evaluating disease severity, particularly in patients with suspected vascular or major organ involvement [6,15]. When used as adjunctive markers, inflammatory indices may assist clinicians in identifying higher-risk patients and guide decisions regarding closer monitoring or further diagnostic evaluation. Importantly, these indices should always be interpreted within the broader clinical context and should not replace clinical judgment, imaging findings, or established disease assessment tools [15,16].

Several limitations should be acknowledged. The retrospective and single-center design may limit generalizability, and the relatively small number of deaths likely reduced the statistical power to detect meaningful associations with mortality. In addition, inflammatory indices were assessed at a single time point, precluding evaluation of longitudinal changes that may better reflect disease activity and progression. Importantly, not all patients were treatment-naïve at the time of laboratory and autoantibody assessments, and prior or ongoing therapies may have influenced inflammatory markers and serological results. Despite these constraints, the study has notable strengths, including a large and well-characterized cohort, a comprehensive comparison of multiple inflammatory indices, and consistent findings across different analytical approaches, collectively supporting the robustness and clinical relevance of the results.

## 5. Conclusions

Our findings show that commonly used inflammatory indices have limited usefulness in predicting mortality in BD. However, these markers—particularly CRP-based composite indices—provide meaningful insights into vascular and major organ involvement. Among them, IBI-NLR and IBI-SII performed best in identifying patients with vascular involvement, while NLR showed modest value for major organ involvement. Across analyses, male sex and elevated ESR consistently emerged as key factors associated with both vascular and major organ involvement, highlighting the role of systemic inflammation in disease severity. In contrast, mortality appeared to be driven mainly by renal dysfunction and inflammatory burden, rather than by inflammatory indices alone. Overall, composite inflammatory indices may be useful supportive tools in clinical assessment, but they should be interpreted cautiously and in conjunction with established clinical and laboratory findings.

## Figures and Tables

**Table 1:** Clinical manifestations, organ involvement and mortality according to gender.

Variables	Total	Female (*n* = 178)	Male (*n* = 266)	*p* Value
Oral aphthae, n (%)	437 (98.4)	174 (97.8)	263 (98.9)	0.446
Genital ulcer, n (%)	329 (74.1)	138 (77.5)	191 (71.8)	0.186
Uveitis, n (%)	183 (41.2)	68 (38.2)	115 (43.2)	0.325
ELL, n (%)	102 (23.0)	47 (26.4)	55 (20.7)	0.169
Acneiform lesions, n (%)	272 (61.4)	102 (57.3)	170 (64.2)	0.164
Vascular involvement, n (%)	90 (20.3)	18 (10.1)	72 (27.1)	0.001
Neurologic involvement, n (%)	41 (9.2)	13 (7.3)	28 (10.5)	0.356
Major organ involvements, n (%)	138 (31.1)	36 (20.2)	102 (38.3)	0.001
Exitus (Mortality), n (%)	13 (2.9)	6 (3.4)	7 (2.6)	0.776

ELL, Erythema nodosum-like lesions.

**Table 2:** Laboratory and inflammatory parameters according to vascular involvement.

Variables	No Vascular Involvement (*n* = 354)	Vascular Involvement (*n* = 90)	*p* Value
WBC (×10^9^/L)	8.04 (0.86–21.01)	9.07 (1.04–27.26)	0.006
Neutrophils (×10^9^/L)	4.93 (0.27–42.00)	5.93 (1.22–25.00)	0.004
Lymphocytes (×10^9^/L)	2.09 (0.13–29.33)	1.89 (0.11–5.03)	0.075
Platelets (×10^9^/L)	286 (29.6–2353)	272 (119–815)	0.527
Hemoglobin (g/dL)	13.8 (4.1–1415)	14.3 (8.6–16.8)	0.093
Creatinine (mg/dL)	0.80 (0.40–31.0)	0.82 (0.38–16.0)	0.018
Urea (mg/dL)	26.0 (0.41–345)	28.0 (0.51–58.0)	0.173
ALT (U/L)	18 (0.18–142)	18.5 (3–194)	0.235
GGT (U/L)	18 (2–177)	25 (1–288)	0.010
Uric acid (mg/dL)	4.4 (0.40–54.0)	4.8 (0.80–54.0)	0.003
CRP (mg/L)	4.62 (0.01–241)	7.83 (0.01–175)	0.001
ESR (mm/h)	17 (1–88)	22.5 (3–143)	0.009
NLR	2.29 (0.12–30.79)	2.87 (0.84–105.25)	0.004
PLR	136.6 (8.8–2102)	147.4 (49.2–5418)	0.319
SII	650.3 (34.6–8220)	733.3 (174.6–52827)	0.044
IBI-NLR	11.91 (0.02–2479.8)	27.65 (0.01–1870.2)	0.0001
IBI-SII	3557 (4.9–952924)	7461 (3.0–1114655)	0.001

WBC, white blood cell count; CRP, C-reactive protein; NLR, neutrophil-to-lymphocyte ratio; PLR, platelet-to-lymphocyte ratio; SII, systemic immune-inflammation index; IBI, inflammatory burden index; ALT, alanine aminotransferase; GGT, gamma-glutamyl transferase.

**Table 3:** Laboratory and inflammatory parameters according to major organ involvement.

Variables	No Major Organ Involvement (*n* = 306)	Major Organ Involvement (*n* = 138)	*p* Value
WBC (×10^9^/L)	8.04 (0.86–21.01)	8.67 (1.04–27.26)	0.003
Neutrophils (×10^9^/L)	4.93 (0.27–42.00)	5.40 (1.22–25.00)	0.010
Lymphocytes (×10^9^/L)	2.09 (0.13–29.33)	2.03 (0.11–5.03)	0.226
Platelets (×10^9^/L)	286.5 (29.6–2353)	263 (119–815)	0.106
Hemoglobin (g/dL)	13.8 (4.1–17.6)	14.1 (4.6–1415)	0.401
Creatinine (mg/dL)	0.79 (0.40–31.0)	0.82 (0.38–27.0)	0.001
Urea (mg/dL)	26.0 (0.41–345)	29.0 (0.51–85.0)	0.004
ALT (U/L)	18 (2–142)	19 (0.18–194)	0.141
GGT (U/L)	17 (2–177)	25 (1–288)	0.005
Uric acid (mg/dL)	4.4 (0.40–49.0)	4.8 (0.80–54.0)	0.010
CRP (mg/L)	4.83 (0.04–241.0)	6.61 (0.01–175.0)	0.136
ESR (mm/h)	17 (1–88)	21.5 (1–143)	0.030
NLR	2.30 (0.12–30.79)	2.66 (0.76–105.25)	0.017
PLR	137.8 (8.8–2102)	136.7 (39.3–5418)	0.911
SII	660.6 (34.6–8220)	695.7 (161.3–52827)	0.234
IBI-NLR	12.19 (0.06–2479.8)	20.69 (0.01–1870.2)	0.010
IBI-SII	3591 (44.1–95292)	5723 (3–1114655)	0.036

WBC, white blood cell count; CRP, C-reactive protein; NLR, neutrophil-to-lymphocyte ratio; PLR, platelet-to-lymphocyte ratio; SII, systemic immune-inflammation index; IBI, inflammatory burden index; ALT, alanine aminotransferase; GGT, gamma-glutamyl transferase.

**Table 4:** Laboratory and inflammatory parameters according to mortality.

Variables	Alive (*n* = 431)	Deceased (*n* = 13)	*p* Value
WBC (×10^9^/L)	8.10 (0.86–27.26)	8.80 (4.46–15.92)	0.246
Neutrophils (×10^9^/L)	5.01 (0.27–42.00)	5.89 (2.34–11.58)	0.285
Lymphocytes (×10^9^/L)	2.07 (0.11–29.33)	2.43 (1.28–4.47)	0.268
Platelets (×10^9^/L)	284 (29.6–2353)	298 (162–529)	0.559
Hemoglobin (g/dL)	13.9 (4.1–1415)	12.2 (10.0–16.6)	0.110
Creatinine (mg/dL)	0.80 (0.38–31.0)	1.00 (0.49–27.0)	0.007
Urea (mg/dL)	26.5 (0.41–345)	28.0 (14–54)	0.551
ALT (U/L)	18 (0.18–194)	16 (8–38)	0.329
GGT (U/L)	19 (1–288)	12 (6–116)	0.185
Uric acid (mg/dL)	4.6 (0.40–54.0)	3.0 (0.64–6.60)	0.001
CRP (mg/L)	5.40 (0.01–241)	5.60 (3.08–34.0)	0.980
ESR (mm/h)	18 (1–143)	32 (9–88)	0.003
NLR	2.36 (0.12–105.25)	2.30 (1.29–5.48)	0.994
PLR	137.7 (8.8–5418)	125.4 (49.2–263.2)	0.636
SII	673.5 (34.6–52827)	795.1 (293.5–2237.4)	0.780
IBI-NLR	13.50 (0.01–2479.8)	13.51 (4.26–114.3)	0.810
IBI-SII	3983 (3.0–1114655)	4552 (1269–38163)	0.684

WBC, white blood cell count; CRP, C-reactive protein; NLR, neutrophil-to-lymphocyte ratio; PLR, platelet-to-lymphocyte ratio; SII, systemic immune-inflammation index; IBI, inflammatory burden index; ALT, alanine aminotransferase; GGT, gamma-glutamyl transferase.

**Table 5:** Receiver Operating Characteristic (ROC) Analysis of Inflammatory Indices in Behçet Disease.

Inflammatory Indexes	Mortality AUC (95% CI)	*p* Value	Major Organ Involvement AUC (95% CI)	*p* Value	Vascular Involvement AUC (95% CI)	*p* Value
NLR (NEU/LYM)	0.501 (0.335–0.666)	0.994	0.571 (0.512–0.629)	0.017	0.597 (0.528–0.666)	0.004
PLR	0.462 (0.328–0.595)	0.636	0.503 (0.443–0.564)	0.911	0.534 (0.463–0.605)	0.319
SII	0.523 (0.346–0.700)	0.780	0.535 (0.476–0.595)	0.234	0.569 (0.498–0.639)	0.044
IBI-NLR (CRP × NLR)	0.520 (0.389–0.650)	0.810	0.576 (0.516–0.636)	0.010	0.624 (0.555–0.692)	<0.001
IBI-SII (CRP × SII)	0.533 (0.408–0.659)	0.684	0.562 (0.502–0.622)	0.036	0.609 (0.539–0.679)	0.001

Abbreviations: AUC, area under the receiver operating characteristic curve; CRP, C-reactive protein; NLR (NEU/LYM), neutrophil-to-lymphocyte ratio; PLR, platelet-to-lymphocyte ratio; SII, systemic immune-inflammation index; IBI, inflammatory burden index.

**Table 6:** ROC-Derived Optimal Cut-off Values of Inflammatory Indices According to Clinical Outcomes.

Markers	Outcomes	Cut-Off (≥)	Sensitivity	Specificity	Youden
NLR (NEU/LYM)	Mortality	1.35–1.37	0.846	0.102	~0.12
	Major organ involvement	≈1.35	0.920	0.114	0.034
	Vascular involvement	≈1.35	0.933	0.114	0.047
PLR	Mortality	107.8–108.2	0.846	0.269	~0.12
	Major organ involvement	≈107.8	0.696	0.269	−0.035
	Vascular involvement	≈107.8	0.700	0.278	−0.022
SII	Mortality	358–361	0.846	0.148	~0.13
	Major organ involvement	≈358–361	0.848	0.147	−0.005
	Vascular involvement	≈358–361	0.856	0.147	0.003
IBI-NLR	Mortality	4.26–4.28	0.923	0.100	~0.12
	Major organ involvement	≈4.9–5.1	0.877	0.154	0.031
	Vascular involvement	≈4.9–5.1	0.922	0.154	0.076
IBI-SII (CRP × SII)	Mortality	1.816–1.828	0.846	0.278	~0.12
	Major organ involvement	≈1.816–1.828	0.761	0.294	0.055
	Vascular involvement	≈1.816–1.828	0.846	0.278	0.124

Abbreviations: AUC, area under the receiver operating characteristic curve; CRP, C-reactive protein; NLR (NEU/LYM), neutrophil-to-lymphocyte ratio; PLR, platelet-to-lymphocyte ratio; SII, systemic immune-inflammation index; IBI, inflammatory burden index.

**Table 7:** Univariate and Multivariable Logistic Regression Analysis of Factors Associated With Mortality.

Variables	Univariate OR (95% CI)	*p* Value	Multivariable OR (95% CI)	*p* Value
Gender (M/F)	0.775 (0.256–2.345)	0.652		
Vascular involvement	0.709 (0.154–3.256)	0.658	—	—
Major organ involvement	0.394 (0.086–1.804)	0.230	—	—
Uric acid	0.352 (0.223–0.555)	<0.001	0.454 (0.278–0.741)	0.002
Creatinine	1.171 (1.088–1.259)	<0.001	1.086 (1.001–1.179)	0.048
ESR (ESH)	1.026 (1.006–1.047)	0.010	1.023 (1.000–1.046)	0.046
Hemoglobin	0.815 (0.630–1.054)	0.119	—	—
Platelet count	1.001 (0.998–1.003)	0.659	—	—
NLR (NEU/LYM)	0.949 (0.743–1.212)	0.674	—	—
PLR	0.997 (0.988–1.005)	0.444	—	—
SII (per 100 units)	0.994 (0.951–1.039)	0.805	—	—
IBI-NLR (per 100 units)	0.718 (0.299–1.723)	0.458	—	—
IBI-SII (CRP × SII per 1000 units)	0.991 (0.965–1.017)	0.483	—	—

OR, odds ratio; CI, confidence interval; ESR (ESH), erythrocyte sedimentation rate; NLR, neutrophil-to-lymphocyte ratio; PLR, platelet-to-lymphocyte ratio; SII, systemic immune-inflammation index; IBI-NLR, inflammation-based index–neutrophil-to-lymphocyte ratio; IBI-SII, inflammation-based index–systemic immune-inflammation index; CRP, C-reactive protein.

**Table 8:** Univariate and Multivariable Logistic Regression Analysis of Factors Associated With Vascular Involvement.

Variables	Univariate OR (95% CI)	*p* Value	Multivariable OR (95% CI)	*p* Value
Gender (M/F)	2.453 (1.578–3.815)	0.0001	3.223 (1.833–5.668)	0.0001
Uric acid	1.026 (0.987–1.066)	0.195	—	—
Creatinine	0.986 (0.906–1.074)	0.749	—	—
ESR (ESH)	1.018 (1.007–1.029)	0.001	1.013 (1.002–1.024)	0.018
Platelet count	1.000 (0.998–1.002)	0.928	—	—
Hemoglobin	0.998 (0.989–1.008)	0.721	—	—
NLR (NEU/LYM)	1.082 (1.013–1.155)	0.019	—	—
PLR	1.001 (1.000–1.002)	0.079	—	—
SII (per 100 units)	1.023 (1.003–1.044)	0.027	—	—
IBI-NLR (per 100 units)	1.106 (1.023–1.195)	0.011		
IBI-SII (CRP × SII per 1000 units)	1.003 (1.001–1.005)	0.007	1.001 (0.999–1.004)	0.295

OR, odds ratio; CI, confidence interval; ESR (ESH), erythrocyte sedimentation rate; NLR, neutrophil-to-lymphocyte ratio; PLR, platelet-to-lymphocyte ratio; SII, systemic immune-inflammation index; IBI-NLR, inflammation-based index–neutrophil-to-lymphocyte ratio; IBI-SII, inflammation-based index–systemic immune-inflammation index; CRP, C-reactive protein.

**Table 9:** Univariate and Multivariable Logistic Regression Analysis of Factors Associated With Major Organ Involvement.

Variables	Univariate OR (95% CI)	*p* Value	Multivariable OR (95% CI)	*p* Value
Gender (M/F)	2.453 (1.578–3.815)	0.001	2.429 (1.554–3.797)	0.001
Uric acid	1.032 (0.993–1.074)	0.109	—	—
Creatinine	1.001 (0.936–1.070)	0.979	—	—
ESR (ESH)	1.013 (1.004–1.023)	0.007	1.011 (1.001–1.023)	0.039
Platelet count	1.000 (0.998–1.002)	0.928	—	—
Hemoglobin	0.998 (0.989–1.008)	0.721	—	—
NLR (NEU/LYM)	1.069 (1.003–1.138)	0.039	—	—
PLR	1.001 (1.000–1.002)	0.158	—	—
SII (per 100 units)	1.020 (1.000–1.040)	0.051	—	—
IBI-NLR (per 100 units)	1.073 (0.995–1.157)	0.067	—	—
IBI-SII (CRP × SII per 1000 units)	1.002 (1.000–1.004)	0.039	1.001 (0.999–1.003)	0.527

OR, odds ratio; CI, confidence interval; ESR (ESH), erythrocyte sedimentation rate; NLR, neutrophil-to-lymphocyte ratio; PLR, platelet-to-lymphocyte ratio; SII, systemic immune-inflammation index; IBI-NLR, inflammation-based index–neutrophil-to-lymphocyte ratio; IBI-SII, inflammation-based index–systemic immune-inflammation index; CRP, C-reactive protein.

## Data Availability

All data can be made available by the corresponding author upon request.

## References

[1] Sakane T., Takeno M., Suzuki N., Inaba G. (1999). Behçet’s disease. New Engl. J. Med..

[2] Yazici H., Seyahi E., Hatemi G., Yazici Y. (2018). Behçet syndrome: A contemporary view. Nat. Rev. Rheumatol..

[3] Davatchi F., Chams-Davatchi C., Shams H., Shahram F., Nadji A., Akhlaghi M., Faezi T., Ghodsi Z., Abdollahi B.S., Ashofteh F. (2017). Behçet’s disease: Epidemiology, clinical manifestations, and diagnosis. Expert Rev. Clin. Immunol..

[4] Davatchi F., Assaad-Khalil S., Calamia K., Crook J., Sadeghi-Abdollahi B., Schirmer M., Tzellos T., Zouboulis C., Akhlagi M., International Team for the Revision of the International Criteria for Behçet’s Disease (ITR-ICBD) (2014). The International Criteria for Behçet’s Disease (ICBD): A collaborative study of 27 countries on the sensitivity and specificity of the new criteria. J. Eur. Acad. Dermatol. Venereol..

[5] Guthrie G.J.K., Charles K.A., Roxburgh C.S.D., Horgan P.G., McMillan D.C., Clarke S.J. (2013). The systemic inflammation-based neutrophil–lymphocyte ratio: Experience in patients with cancer. Crit. Rev. Oncol./Hematol..

[6] Yang R., Chang Q., Meng X., Gao N., Wang W. (2018). Prognostic value of systemic immune-inflammation index in cancer: A meta-analysis. J. Cancer.

[7] Fest J., Ruiter T.R., Groot Koerkamp B., Rizopoulos D., Ikram M.A., van Eijck C.H.J., Stricker B.H. (2018). The neutrophil-to-lymphocyte ratio is associated with mortality in the general population. Eur. J. Epidemiol..

[8] Zahorec R. (2001). Ratio of neutrophil to lymphocyte counts—Rapid and simple parameter of systemic inflammation and stress in critically ill. Bratisl. Lekárske Listy.

[9] Gasparyan A.Y., Ayvazyan L., Mukanova U., Yessirkepov M., Kitas G.D. (2019). The platelet-to-lymphocyte ratio as an inflammatory marker in rheumatic diseases. Ann. Lab. Med..

[10] Hu B., Yang X.-R., Xu Y., Sun Y.-F., Sun C., Guo W., Zhang X., Wang W.-M., Qiu S.-J., Zhou J. (2014). Systemic immune-inflammation index predicts prognosis of patients after curative resection for hepatocellular carcinoma. Clin. Cancer Res..

[11] Kural-Seyahi E., Fresko I., Seyahi N., Ozyazgan Y., Mat C., Hamuryudan V., Yurdakul S., Yazici H. (2003). The long-term mortality and morbidity of Behçet syndrome: A 2-decade outcome survey of 387 patients followed at a dedicated center. Medicine.

[12] Wu J., Tan W., Chen L., Huang Z., Mai S. (2018). Clinicopathologic and prognostic significance of C-reactive protein/albumin ratio in patients with solid tumors: An updated systemic review and meta-analysis. Oncotarget.

[13] Seyahi E. (2019). Behçet’s disease: How to diagnose and treat vascular involvement. Best Pract. Res. Clin. Rheumatol..

[14] Saadoun D., Wechsler B., Desseaux K., Le Thi Huong D., Amoura Z., Resche-Rigon M., Cacoub P., Piette J.C. (2010). Mortality in Behçet’s disease. Arthritis Rheum..

[15] Pearson T.A., Mensah G.A., Alexander R.W., Anderson J.L., Cannon R.O., Criqui M., Fadl Y.Y., Fortmann S.P., Hong Y., Myers G.L. (2003). Markers of inflammation and cardiovascular disease: Application to clinical and public health practice. Circulation.

[16] Furman D., Campisi J., Verdin E., Carrera-Bastos P., Targ S., Franceschi C., Ferrucci L., Gilroy D.W., Fasano A., Miller G.W. (2019). Chronic inflammation in the etiology of disease across the life span. Nat. Med..

